# Fabricating a designer capsule phase microextraction platform based on sol–gel Carbowax 20M-zwitterionic ionic liquid composite sorbent for the extraction of lipid-lowering drugs from human urine samples

**DOI:** 10.1007/s00604-023-05998-3

**Published:** 2023-10-05

**Authors:** Argyroula Kechagia, Natalia Manousi, Abuzar Kabir, Kenneth G. Furton, Constantinos K. Zacharis

**Affiliations:** 1 Laboratory of Pharmaceutical Analysis, Department of Pharmaceutical Technology, School of Pharmacy, Aristotle University of Thessaloniki, 54124 Thessaloniki, Greece; 2 Department of Chemistry and Biochemistry, International Forensic Research Institute, Florida International University, Miami, FL 33131 USA

**Keywords:** Capsule phase microextraction, Designer sorbents, Ionic liquids, Statins, HPLC, Urine analysis

## Abstract

A sol–gel Carbowax 20 M/3-[(3-Cholamidopropyl) dimethyl ammonio]-1-propanesulfonate composite sorbent-based capsule phase microextraction device has been fabricated and characterized for the determination of four statins (pravastatin, rosuvastatin, pitavastatin, and atorvastatin) in human urine. The presence of ionizable carboxyl functional groups in statins requires pH adjustment of the sample matrix to ensure that the target molecules are in their protonated form (pH should be 2 units below their pK_a_ values) which not only is cumbersome but also risks unintended contamination of the sample. This challenge was addressed by introducing zwitterionic ionic liquid in addition to neutral, polar Carbowax 20 M polymer in the sol–gel-derived composite sorbent. As such, the composite zwitterionic multi-modal sorbent can simultaneously extract neutral, cationic, and anionic species. This particular attribute of the composite sorbent eliminates the necessity of the matrix pH adjustment and consequently simplifies the overall sample preparation workflow. Various experimental parameters such as the sample amount, extraction time, salt addition, stirring rate, and elution solvent type that may affect the extraction performance of the statins were investigated using a central composite design and the one-parameter-at-a-time approach. The analytes and the internal standard were separated on a C_18 _column with gradient elution using phosphate buffer (20 mM, pH 3) and acetonitrile as mobile phase. The analytes were detected at 237 nm. The method was validated, and linearity was observed in the range 0.10–2.0 μg mL^−1^ for all compounds. The method precision was better 9.9% and 10.4% for intra-day and inter-day, respectively, while the relative recoveries were acceptable, ranging between 83.4 and 116% in all cases. Method greenness was assessed using the ComplexGAPI index. Finally, the method’s applicability was demonstrated in the determination of the statins in authentic human urine after oral administration of pitavastatin and rosuvastatin-containing tablets.

**Graphical abstract:**

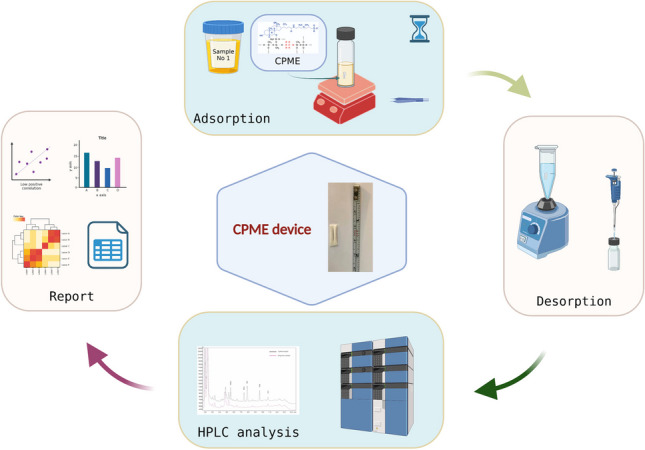

**Supplementary Information:**

The online version contains supplementary material available at 10.1007/s00604-023-05998-3.

## Introduction

Statins comprise a class of lipid-lowering drugs that are widely used for the reduction of blood cholesterol and triglyceride levels in patients suffering from cardiovascular complications and patients who are at increased risk for atherosclerosis development [1]. These drugs are competitive inhibitors of 3-hydroxy-3-methylglutaryl coenzyme A reductase, an enzyme that can catalyze a previous and limiting stage of cholesterol biosynthesis [2]. Although statins are individually used during therapy, the strategies that enable their simultaneous monitoring in real samples are very useful for assessment in quality control. Currently, the high importance of statins in clinical use demands the development of sensitive and accurate analytical methods for their monitoring in biological fluids [3]. For the determination of statins, LC–MS/MS systems are typically employed due to their high sensitivity and selectivity, while simpler and cost-effective HPLC–UV approaches have been used as well. The bioanalytical perspectives of statins have been critically reviewed by Patel et al. [4]. Due to the complex nature of biological matrices, sample preparation is normally required prior to their instrumental determination.

The traditional sample preparation techniques that are widely used in bioanalysis (i.e., protein precipitation, solid-phase extraction, and liquid–liquid extraction) show multiple drawbacks such as increased consumption of chemicals and laborious steps. Thus, sample preparation is considered to be the bottleneck of analytical chemistry. Currently, the development of environmentally friendly extraction techniques is at the forefront of research. In this context, various liquid-phase and sorbent-based miniaturized extraction approaches have been proposed [5]. Classical examples of sorbent-based techniques include solid-phase microextraction (SPME), stir bar sorptive extraction (SBSE), magnetic solid-phase extraction, dispersive solid-phase extraction, and fabric phase sorptive extraction (FPSE), and capsule phase microextraction (CPME) [6–10]. These techniques have been developed in accordance with the requirements of Green Analytical Chemistry (GAC) [11]. Tailor-made materials like molecularly imprinted polymers (MIPs) [12, 13], MOFs, magnetic nanoparticles, hyper-porous materials, and carbon allotropes [14] in combination with the above-mentioned microextraction techniques offer a diversified toolkit for cheap, fast, and environmentally friendly sample preparation.

Capsule phase microextraction was recently introduced by Kabir and Furton [15]. In CPME, specially designed microextraction capsules are used which consist of the following parts: (a) a permeable microporous polypropylene membrane, (b) a sol–gel hybrid inorganic–organic sorbent, and (c) a cylindrical magnet. In this case, the porous membrane acts as the “filtration mechanism” of the capsule, while the incorporation of the magnet integrates the stirring mechanism. Furthermore, sol–gel sorbents can provide high extraction efficiency toward the desired compounds due to the inherent material properties of these composite sorbents. Until now, a wide variety of sol–gel sorbent integrated CPME capsules have been designed and used for the extraction of analytical and bioanalytical analytes of interest. Typical examples of sorbents that have been used during the development of microextraction capsules include sol–gel octadecyl [16], sol–gel poly(tetrahydrofuran) [17], sol–gel poly(ethylene glycol) [18], sol–gel Carbowax 20 M [19], and mixed-mode ion-exchange sorbents [20]. Sol–gel technology can provide strong retention of the coating onto the substrate due to chemical bonding. The inherent porous structure of the obtained sorbent favors the rapid mass transfer of the desired compounds onto the sorbent, thus reducing the extraction equilibrium time [21].

Currently, great attention is also being paid to the development and application of new materials in sample preparation. In recent years, ionic liquids (ILs) have attracted the attention of many researchers working in the field of sample preparation due to their unique physicochemical properties [22]. ILs are organic salts composed of bulky organic cations and small anions of different natures, and they are molten at temperatures below 100 °C. They are characterized by high thermal and chemical stability, preparation simplicity, low vapor pressure, and good affinity for organic and inorganic analytes. An important benefit of ILs is the possibility to tune their properties, and thus, ILs have been characterized as “designer solvents” [23]. They have gained a lot of attention both as extraction solvents in liquid-phase microextraction and for the functionalization of sorbents in sorbent-based microextraction [24].

To the best of our knowledge, the exploration of ILs for the fabrication of task-specific CPME devices has not been reported. Task-specific materials exhibit high specificity toward the target analyte(s). The most important benefit of these materials includes the enhancement of selectivity which is a significant issue in bioanalysis since biological matrices often contain a significant source of interferences [23].

In this work, a designer sample preparation platform based on sol–gel Carbowax 20 M/ IL composite sorbent for the CPME of statins from human urine is presented. The obtained microextraction devices combine the benefits of selectivity and high extraction efficiency of sol–gel Carbowax 20 M/IL composite sorbent and the handling simplicity and inherent benefits of CPME devices. Compared to the conventional C_18 _material, the fabricated material provided better extraction efficiency. The novel microextraction capsules were characterized by scanning electron microscopy (SEM) and Fourier-transform infrared spectroscopy (FT-IR). The optimization of the sample preparation step was conducted using both a face-centered central composite design (FC-CCD) and the one-factor-at-a-time (OFAT) approach. Method validation was conducted in accordance with the FDA guidelines for bioanalytical methodologies, and the ComplexGAPI index was employed to assess method greenness. The CPME-HPLC–UV protocol was employed for the quantitation of the target analytes in human urine samples from patients who underwent statin therapy.

## Experimental

### Materials, reagents, and solutions

Atorvastatin, pitavastatin, rosuvastatin, pravastatin (> 98.0%), and emamectin benzoate (≥ 98.0%) were purchased from Sigma-Aldrich (St. Louis, MO, USA). All organic solvents were of HPLC grade, and they were obtained from Honeywell (New Jersey, USA). Milli-Q water was produced by a B30 purification system (Adrona SIA, Riga, Latvia).

The stock solutions of each analyte (1000 μg mL^−1^) were prepared in methanol. An emamectin benzoate solution (1000 μg mL^−1^) (used as internal standard, ISTD) was made in acetonitrile. All solutions were kept at 4 °C. Daily, working solutions were made in water from the respective stock solutions.

Artificial urine was prepared according to Brooks and Keevil [25] and used in the method development. In brief, urea (5 g), NaCl (2.6 g), NaHCO_3_ (1.05 g), NH_4_Cl (0.65 g), lactic acid (0.05 g), K_2_HPO_4_ (0.6 g), MgSO_4_·7H_2_O (0.25 g), CaCl_2_·2H_2_O (0.19 g), KH_2_PO_4_ (0.48 g), Na_2_SO_4_·10H_2_O (1.6 g), and citric acid (0.2 g) were dissolved in 500 mL water. Accordingly, the pH of the solution was adjusted to 7.0 using HCl.

Microextraction capsules were fabricated using Accurel® porous propylene capillary membranes obtained from 3 M Inc. (St. Paul, MN, USA). Cylindrical magnets used in the CPME device fabrication were purchased from K&J Magnetics Inc. (Pipersville, PA, USA). Analytical grade methanol, methylene chloride, NH_4_OH (28%), and HCl (37%), as well as Carbowax 20 M, were purchased from Fisher Scientific (Milwaukee, WI, USA). Methyl trimethoxysilane (MTMS), 3-[(3-Cholamidopropyl) dimethyl ammonio]-1-propanesulfonate and tetramethyl orthosilicate (TMOS) (> 98.0%) were obtained from Sigma-Aldrich (St. Louis, MO, USA).

### Instrumentation

The analytical method used for the separation and quantification of statins in the urine samples was developed in-house. A Shimadzu 2010A HPLC–UV system (Kyoto, Japan) and a Supelco C_18_ analytical column (150 mm × 4.0 mm, 5 μm, Supelco Inc, Bellefonte, PA, USA) were employed throughout this study. The temperature of the column was set at 30 °C. A mixture of (A) phosphate buffer 20 mM (pH adjusted to 3 with H_3_PO_4_) and (B) acetonitrile was used as the mobile phase. The flow rate of which was 1.0 mL min^−1^. Gradient elution of the target analytes was performed as follows: The initial mobile phase composition was 75:25, A/B v/v (constant for 1.5 min). Then, the composition changed to 25:75 A/B v/v at 10 min (constant until 11 min). The mobile phase returned to the initial composition at 12 min, and the system was further equilibrated until 22 min. The detection and quantitation of the statins and the ISTD were performed at 237 nm. The injection volume of the sample volume into the chromatographic system was 10 μL.

### Sample collection and pretreatment

All urine samples were provided from healthy individuals after providing full information about the nature of the study. All volunteers provided their written consent. During method development, drug-free samples were used. Initially, 450 μL of the human urine sample was mixed with 25 μL of Milli-Q water for blank samples or 25 μL of the working solutions containing the target analytes for spiked samples. An aliquot of 25 μL of ISTD solution (20 μg mL^−1^) was also added to the samples prior to the CPME protocol. Due to the inherent characteristics of the capsules, no other pretreatment step (e.g., filtration) is required. The developed and validated CPME-HPLC–UV method was finally used for the analysis of real samples from patients who underwent treatment with rosuvastatin and pitavastatin. Also, in this case, the individuals were fully informed regarding the study, and they provided their written consent.

### CPME protocol

The microextraction protocol was performed in four consecutive steps, namely, activation, adsorption, elution, and cleaning. Antistatic tweezers were used for the handling of the capsules throughout the CPME protocol to avoid contamination.

- Activation: The CPME platform was placed into a glass vial containing 0.5 mL of MeOH for surface activation. After 5 min, the capsule was removed, and it was excessively rinsed with Milli-Q water.
- Adsorption: For the extraction of statins, the capsule was placed into a clean vial containing 500 μL of urine sample. The adsorption process was completed within 33 min under constant stirring at 300 rpm. After this timespan, the capsule was removed from the sample. Capsule cleaning was performed through rinsing with water, followed by wiping with a lint-free tissue.
- Elution: The CPME membrane was placed into an Eppendorf tube, and 500 μL of MeOH was added for the desorption. The capsule was removed after 5 min, and the eluate was analyzed by HPLC–UV.
- Cleaning: Following a complete sample preparation cycle, the CPME platform was washed using the aliquot of MeOH of the activation step. The capsule can be stored and reused when necessary. Under these conditions, the CPME platforms were found to be reusable at least 35 times.

### Preparation of CPME platforms

Fabrication of CPME devices with encapsulated sol–gel Carbowax 20 M/IL composite sorbent involves four distinct steps: (a) fabrication and washing of empty capsule phase microextraction devices; (b) sol solution design, preparation, and optimization; (c) fabrication of the simultaneous surface coating and the monolithic bed of the sol–gel sorbent on the walls of the device as well as in the lumen of the device; (d) aging, conditioning, and washing.

The CPME devices were prepared at a length of 1 cm, considering the relatively low volume of urine samples. To fabricate the CPME devices, Accurel® propylene porous membranes were cut into 1 cm lengths, and they were washed using methylene chloride under ultrasonic irradiation for 30 min, followed by air drying. A cylindrical bar magnet (1/4˝ × 1/16˝) was installed into one 1-cm porous polypropylene tube membrane. Subsequently, one propylene tube containing the cylindrical bar magnet and one empty propylene tube were joined together using impulse heat sealing. As a result, both propylene tube membranes are connected side by side to each other with both ends heat-sealed, and the device is ready for the coating procedure.

The sol solution was prepared through the sequential addition of TMOS, MTMS, organic polymer, methylene chloride, methanol, hydrochloric acid (0.1 M), and ionic liquid in a 50-mL reaction vessel at molar ratio: 1:1:0.02:5:10:8:0.2. The solution was kept at 50 °C for 12 h so that the sol–gel precursors were completely hydrolyzed. Following centrifugation, the supernatant sol solution was placed into a clean 50-mL vessel. To transform the sol solution into the gel, NH_4_OH (1 M) was added to the sol solution dropwise under continuous stirring (800 rpm) at a molar ratio TMOS:NH_4_OH of 1:10. The gelation of the sol solution occurred in 60 min. Because of the reduced viscosity of the sol solution in the beginning, the sol solution freely permeated through the pores of the CPME devices and filled the lumens of the tube with the sol solution. Then, there is an increase in the viscosity of the sol solution after the addition of NH_4_OH, and the sol solution is trapped within the pores of the porous propylene tube to form particles and a monolithic bed within the lumens. Once the sol solution turned into sol–gel, the CPME devices were subjected to aging and conditioning at 50 °C for 24 h. The CPME devices were cleaned and rinsed with a mixed solvent system, methylene chloride:methanol (50:50 v/v), under ultrasonic irradiation within 30 min. During this process, the monolithic bed was trapped within the lumens of the propylene tubes disintegrated into fine particles. Finally, drying was employed at 50 °C for 2 h.

### Method validation

According to the guidelines of the FDA for bioanalytical methods [26], validation of the CPME-HPLC–UV protocol was performed in terms of linearity, accuracy, precision, matrix effect, selectivity, limit of quantification (LOQ), and limit of detection (LOD). Method linearity was assessed using a matrix-matched and an aqueous calibration curve that was constructed within the working range of 0.10–2.0 μg mL^−1^. For this purpose, the peak area of each statin was plotted against the peak area of the ISTD. Triplicate analysis took place for all concentration levels used for the assessment of linearity. As LLOQ, the lowest point of the calibration curve with *S*/*N* > 10 was set, while the LOD was calculated according to the criterion *S*/*N* > 3. The matrix effect (% ME) was assessed in terms of relative error between the slope of the individual matrix-matched regression equation for each statin versus the slope of the respective aqueous regression equation. The evaluation of the method’s sensitivity was performed by analyzing statins-free real urine samples, along with their spiked analogs. The accuracy and precision were studied through the analysis of spiked samples at four different concentration levels, i.e., lower limit of quantification (LLOQ): 0.10 μg mL^−1^; low-quality control level (LQC): 0.25 μg mL^−1^; medium quality control level (MQC): 0.75 μg mL^−1^; and high-quality control level (HQC): 2.0 μg mL^−1^. Five analyses were performed within the same day for the intra-day study, while the inter-day study was performed in terms of triplicate analysis on four consecutive days.

## Results and discussion

### Creation of the designer capsule phase microextraction platform based on sol–gel Carbowax 20 M/zwitterionic ionic liquid composite sorbent

**Fig. 1:**
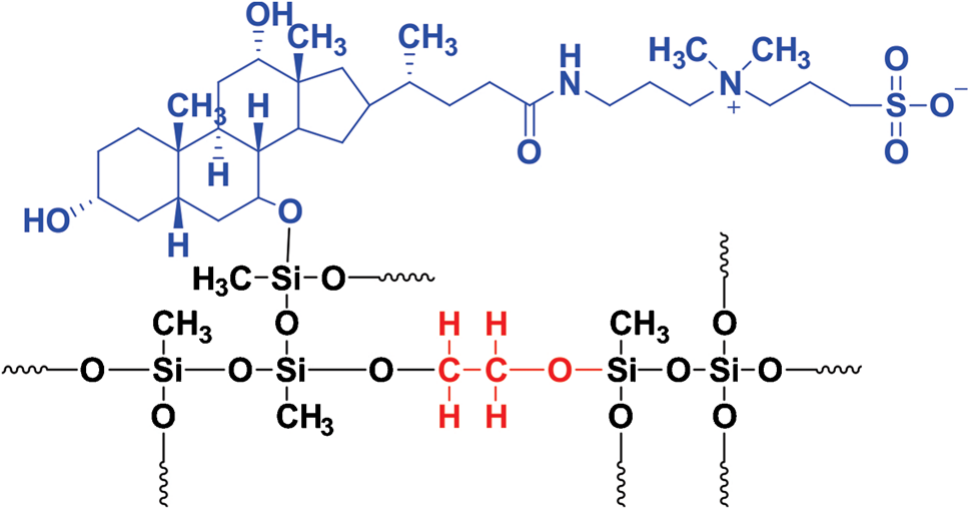
Chemical structure of sol–gel Carbowax 20 M/3-[(3-Cholamidopropyl) dimethyl ammonio]-1-propanesulfonate sorbent (ionic liquid is presented in blue, Carbowax 20 M is presented in red, inorganic precursor is presented in black)

Commercially available sorbents used in solid-phase extraction and solid-phase microextraction perform well when the compounds are either polar or nonpolar. However, when the target analytes include both polar and nonpolar compounds, it poses a great analytical challenge. The challenge becomes seriously complicated when the analytes are ionizable (organic acids or bases). The target analytes in the current study are weak organic bases possessing p*K*_a_ values ranging from 4.0 to 4.3 and log*K*_ow_ values ranging from 2.18 (polar) to 6.36 (nonpolar) (Supplementary Table S1). As a result, a polar, nonpolar, or mixed-mode sorbent alone is not sufficient to simultaneously achieve high extraction recovery of all the target compounds. One possible solution to overcome the impasse is to create “a designer sorbent” that would offer unique selectivity and extractive affinity toward all the target analytes. The concept was materialized by designing a designer sorbent consisting of an organic polymer, Carbowax 20 M (polar polymer), and a zwitterionic ionic liquid (carrying both cation, anion, and hydrophobic backbone). A schematic presentation of the sol–gel Carbowax 20 M/3-[(3-Cholamidopropyl) dimethyl ammonio]-1-propanesulfonate composite sorbent is presented in Fig. 1. Different components of the sol–gel composite materials are shown in different colors: The IL part is presented in green, Carbowax 20 M is presented in red, and sol–gel precursors are presented in black. The methyl pendant groups originated from the methyl trimethoxy silane, a sol–gel precursor, provide a London dispersion type of interaction with the target analytes and serve to modify the hydrophobicity of the composite sorbent so that the sorbent may effectively interact with both polar and nonpolar sorbents. As can be seen, the sol–gel composite sorbent, due to its unique composition, is capable of simultaneously extracting polar, nonpolar, and ionizable compounds via different intermolecular/interionic interactions including dipole–dipole interactions, electrostatic interactions, hydrogen bonding, and London dispersion. It is worth noting that the “designer sorbent” should perform well with other mixes of analytes as the extraction efficiency of the sol–gel designer sorbent primarily depends on the interactions among the analytes and the sorbent via different functional groups, not the individual identity of the analyte. Extraction recovery data presented in Supplementary Fig. S3 demonstrates a clear trend that extraction recovery values are strongly correlated with the logK_ow_ values of the analytes. When the extraction recovery values are compared with the classical sol–gel C_18_ sorbent-based CPME device, all analytes were poorly extracted due to the absence of Carbowax 20 M and zwitterionic ionic liquid. It appears that C_18_, a nonpolar/hydrophobic moiety, did not offer high extraction recovery of the nonpolar analyte atorvastatin. It is fair to hypothesize that hydrophobic interaction was not the dominant interaction in the extraction process. In the case of the designer sol–gel Carbowax 20 M/IL composite sorbent, both the organic polymer and the ionic liquid collectively contribute to the selectivity and extractive affinity toward the target analytes. The sol–gel Carbowax 20 M-zwitterionic ionic liquid composite sorbent used in the current study was prepared using the hydrolytic sol–gel process. Hydrolytic sol–gel synthesis of sorbent typically consists of two steps: (a) hydrolysis of the sol–gel precursors to convert alkoxy functional groups into hydroxyl functional groups; (b) polycondensation of hydroxyl groups of neighboring units to form a three-dimensional network. Acid-catalyzed sol–gel reaction favors hydrolysis and linear growth of the network, taking prolonged periods to form a solid gel. TMOS and MTMOS used in the current sol–gel synthesis have different hydrolysis and polycondensation kinetics. As such, the sol–gel synthesis was carried out in two steps using two catalysts: acid-catalyzed hydrolysis (0.1 M HCl) and base-catalyzed polycondensation (1.0 M NH_4_OH). The application of two catalysts provides better control in the synthesis and results in a sorbent possessing sponge-like porous micro-architecture and high surface area.

An important characteristic of any new sorbent is its maximum analyte adsorption capacity at equilibrium extraction conditions. However, the adsorption capacity of sol–gel Carbowax 20 M-zwitterionic ionic liquid composite sorbent was not estimated because the concentration of the target analytes will always be in trace or ultra-trace level due to the low dosage of the drugs. As such, the sorbent will never be saturated. In case the concentration of the drug (potential drug overdose) exceeds the limit, the sample can be diluted to reduce the concentration for quantitative analysis.

### Unique selectivity of sol–gel Carbowax 20 M-zwitterionic ionic liquid composite sorbent

The selectivity of the designer sorbent was designed and created to maximize the intermolecular/electrostatic interactions between the sorbent and target analytes. Classical sorbents such as polydimethylsiloxane or C_18_ offer only weak Vander Waals force and require matrix pH adjustment to force the ionizable analytes to remain in their neutral state. However, the new sol–gel CW 20 M/IL-based sorbent offers, by design, Vander Waals force, hydrogen bonding, dipole–dipole interaction, and electrostatic interactions simultaneously toward the target analytes. Sol–gel synthesis, unlike pristine polymeric sorbents, allows implanting of numerous functional groups via silane precursors to maximize these analyte-sorbent interactions. On the other hand, the selectivity parameter of any pristine polymer is its inherent characteristics and cannot be modified. As such, sol–gel sorbents offer superior selectivity compared to pristine polymers.

It is true that the presence of diversified interactions may extract some unwanted analytes. But, our method development process has demonstrated that the new sorbent offers unique selectivity toward the analytes and did not extract any interferents from the urine matrix that may interfere with the chromatographic analysis. To compensate for the selectivity lack of any general-purpose sorbent, we have taken advantage of the chromatographic separation technique.

### Characterization of the IL-based CPME platform

The designer sorbent sol–gel Carbowax 20 M/3-[(3-Cholamidopropyl) dimethyl ammonio]-1-propanesulfonate composite sorbent was characterized by SEM and FT-IR.

#### Scanning electron microscopy

**Fig. 2:**
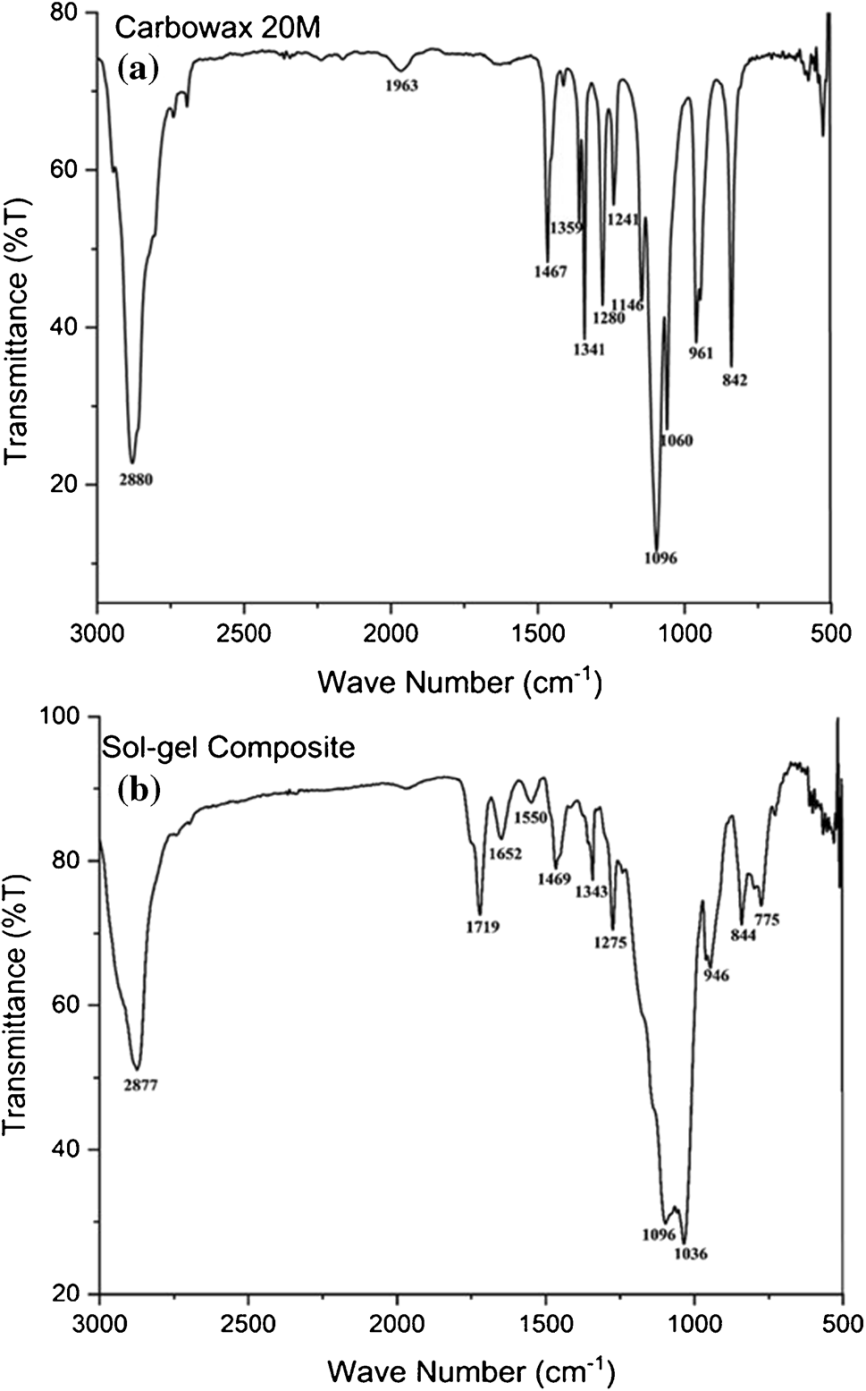
FT-IR spectra of (**a**) pristine Carbowax 20 M polymer; (**b**) sol–gel Carbowax 20 M/3-[(3-Cholamidopropyl) dimethyl ammonio]-1-propanesulfonate sorbent

SEM helps investigate the surface morphology of the substrate, distribution of the particles in pores of the walls of CPME medium, and the particle size distribution of crushed sorbent. Figure 2a–d shows the SEM images. The walls of the polypropylene tubes are porous with a nominal pore size of 0.2 µm. The SEM image of the propylene tube walls presented in Fig. 2a reveals the homogeneously distributed pores that can be used as micro pockets to retain sol–gel microparticles during the sol–gel sorbent synthesis. Figure 2b represents a cross-section of the CPME device. The particles of the sol–gel sorbent that were trapped inside the walls of the CPME device are clearly seen in the image. A circle is made on the wall so that the viewers can focus to see the particles clearly. Figure 2c presents an expanded view of the wall. As it is revealed, the sol–gel sorbent particles vary in their sizes, from nanosized to microsized. The particle size distribution of the crushed particles within the lumens of the CPME device is presented in Fig. 2d. Similar to particles trapped with the pores of the propylene walls, the particles inside the lumens are not homogeneously distributed in their sizes, some particles are large (~ several microns), and some are very small (nanosized). The fine particles of the sol–gel sorbent translate into a very high specific surface area. As a result, an aqueous solution containing the target analytes interacts with the particles rapidly, resulting in a very fast extraction equilibrium.

#### Fourier-transform infrared spectroscopy

**Fig. 3:**
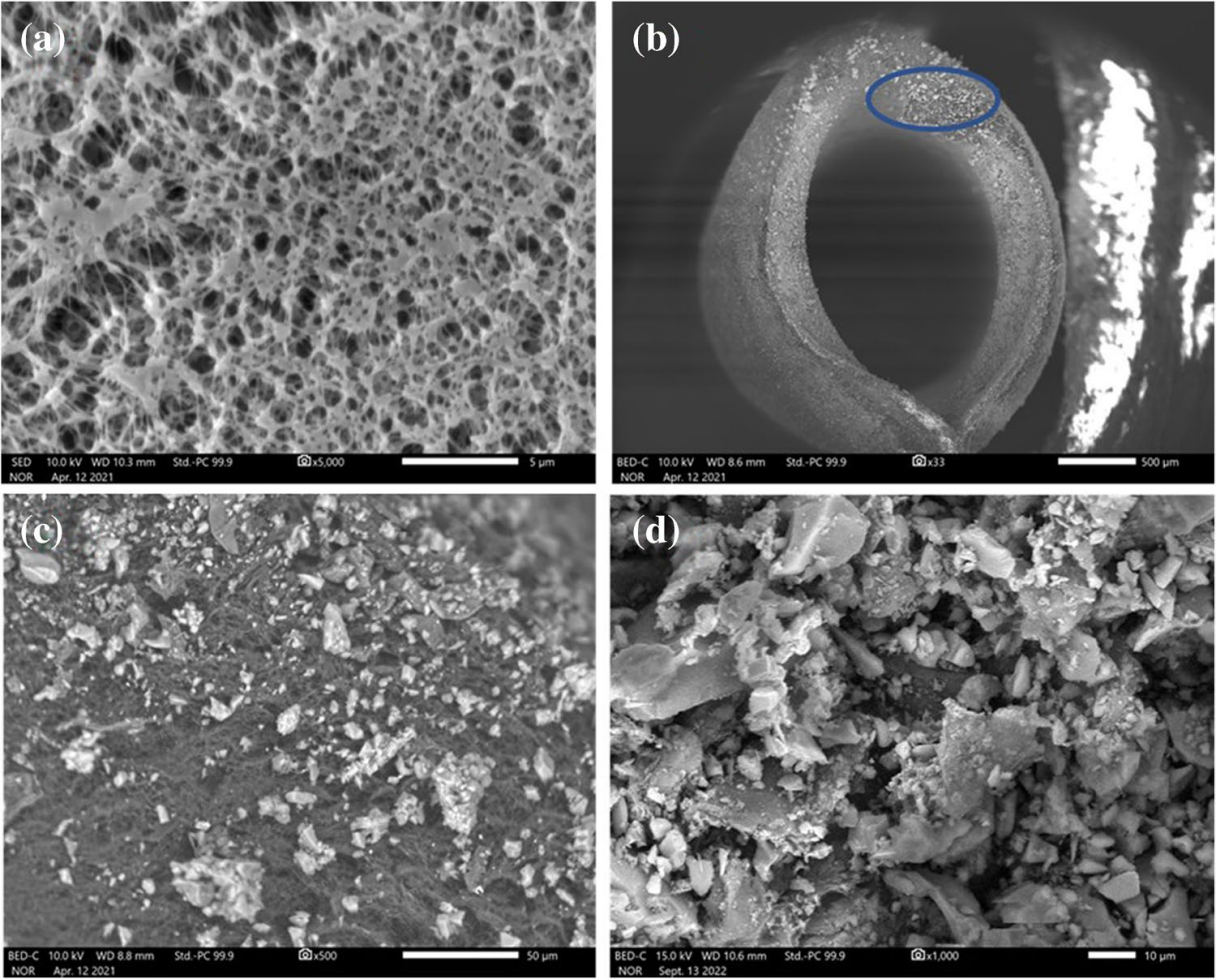
Scanning electron microscopy images of (**a**) porous polypropylene capillary surface prior to sol–gel sorbent coating at 5000 × magnifications; (**b**) a cross-section of sol–gel Carbowax 20 M-3-[(3-Cholamidopropyl) dimethyl ammonio]-1-propanesulfonate sorbent-coated microextraction capsule at 33 × magnifications; (**c**) distribution of sol–gel sorbent particles distributed within the walls of microextraction capsule; (**d**) crushed sol–gel sorbent particles obtained from the monolithic bed created within the lumen of the microextraction capsule

FT-IR spectroscopy plays an instrumental role in establishing the presence of different functional groups in different building blocks of sol–gel sorbent as well as in the sol–gel composite sorbent. The FT-IR spectra of the major building block, Carbowax 20 M and sol–gel Carbowax 20 M/3-[(3-Cholamidopropyl) dimethyl ammonio]-1-propanesulfonate composite sorbent, are presented in Fig. 3a, b, respectively. The FT-IR spectra of MTMS and ionic liquid are presented in Supplementary Fig. S1a,b, respectively.

Characteristics absorption bands of Carbowax 20 M polymer include C-O, C–C stretching, CH_2_ rocking at 842 cm^−1^; CH_2_ rocking, CH_2_ twisting at 961 cm^−1^; C-O stretching, CH_2_ rocking at 1146 cm^−1^; CH_2_ twisting at 1241 cm^−1^ and 1280 cm^−1^; CH_2_ wagging at 1341 cm^−1^; and CH_2_ scissoring at 1467 cm^−1^ [27].

The characteristic absorption bands in the MTMS FT-IR spectra (Supplementary Fig. S1a) include the Si–O-CH band at 2949 cm^−1^ [28]. The bands of 1269 cm^−1^, 845 cm^−1^, and 795 cm^−1^ belong to the Si-CH_3_ bond [29].

As for the FT-IR spectra of the ionic liquid, major absorption bands include bands at 1176 cm^−1^, 1037 cm^−1^, and 1645 cm^−1^ belonging to S–O, S-OH, and C = O bonds, respectively [30].

Many absorption bands simultaneously appear in the FT-IR spectra of the individual building blocks as well as in the sol–gel Carbowax 20 M/3-[(3-Cholamidopropyl) dimethyl ammonio]-1-propanesulfonate composite sorbent that strongly suggests the successful incorporation of the building blocks in the gel composite sorbent.

#### Storage lifetime of sol–gel Carbowax 20 M-zwitterionic ionic liquid composite sorbent

Capsule Phase Microextraction devices are built using porous polypropylene tubular membranes. The sol–gel silica-based composite sorbent was prepared using patented sol–gel technology. Silica-based materials are very robust and durable. As such, it is fair to anticipate long self-life for CPME devices. However, since CPME devices are considered as laboratory consumable, their self-life has not been investigated.

### Optimization of the CPME protocol

The optimization of the CPME method was performed using artificial urine samples spiked at a concentration level of 1.0 μg mL^−1^. Initially, the sample volume, extraction time, and stirring rate were optimized using the Box-Behnken design. Following the selection of the optimum parameters, the “one-factor-at-a- time” (OFAT) approach was used for the optimization of the main chemical factors (i.e., ionic strength, sample pH) and desorption conditions.

#### Optimization by Box-Behnken design

A Box-Behnken design (BBD) has been employed to optimize the sample volume (factor A), stirring rate (factor B), and extraction time (factor C) involving 18 runs. Supplementary Table S2 summarizes the examined parameters, their levels, and the experimental domain created using Design-Expert 13 software (Stat-Ease Inc®, Minneapolis, MN, USA). Analysis of variance (ANOVA) was used for model validation, where the non-significant factors (*p* > 0.05) were excluded using the “backward elimination” process (Supplementary Tables S3–S6). The calculated *R*^2^ and the adjusted *R*^2^ were higher than 0.7391 and 0.7043, respectively, for all compounds, indicating that the models adequately explain the response. The validity of the models was evaluated through the plot of the residuals in comparison with the predicted values and the normal probability plot of residuals (Supplementary Fig. S2). The monotonous scattering of data around the line reveals the good correlation between the actual and the predicted responses. Supplementary Fig. S3 shows the contour plots and the 3D response surface for all analytes. Τhe highest extraction efficiency of all drugs was recorded at lower sample volumes and higher extraction times. Derringer’s desirability function was used to find the optimum conditions by setting the stirring rate (non-significant) value at 300 rpm while the extraction time was minimized. A desirability of 0.7136 was achieved, and the optimum values were estimated to be 500 min and 33 min (rounded) for sample volume and extraction time, respectively. The experimental conditions were confirmed by performing the extraction in six repetitions. The variation of the predicted values and the experimentally found values were < 8%, which was considered satisfactory.

#### Study of sample pH and ionic strength

**Fig. 4:**
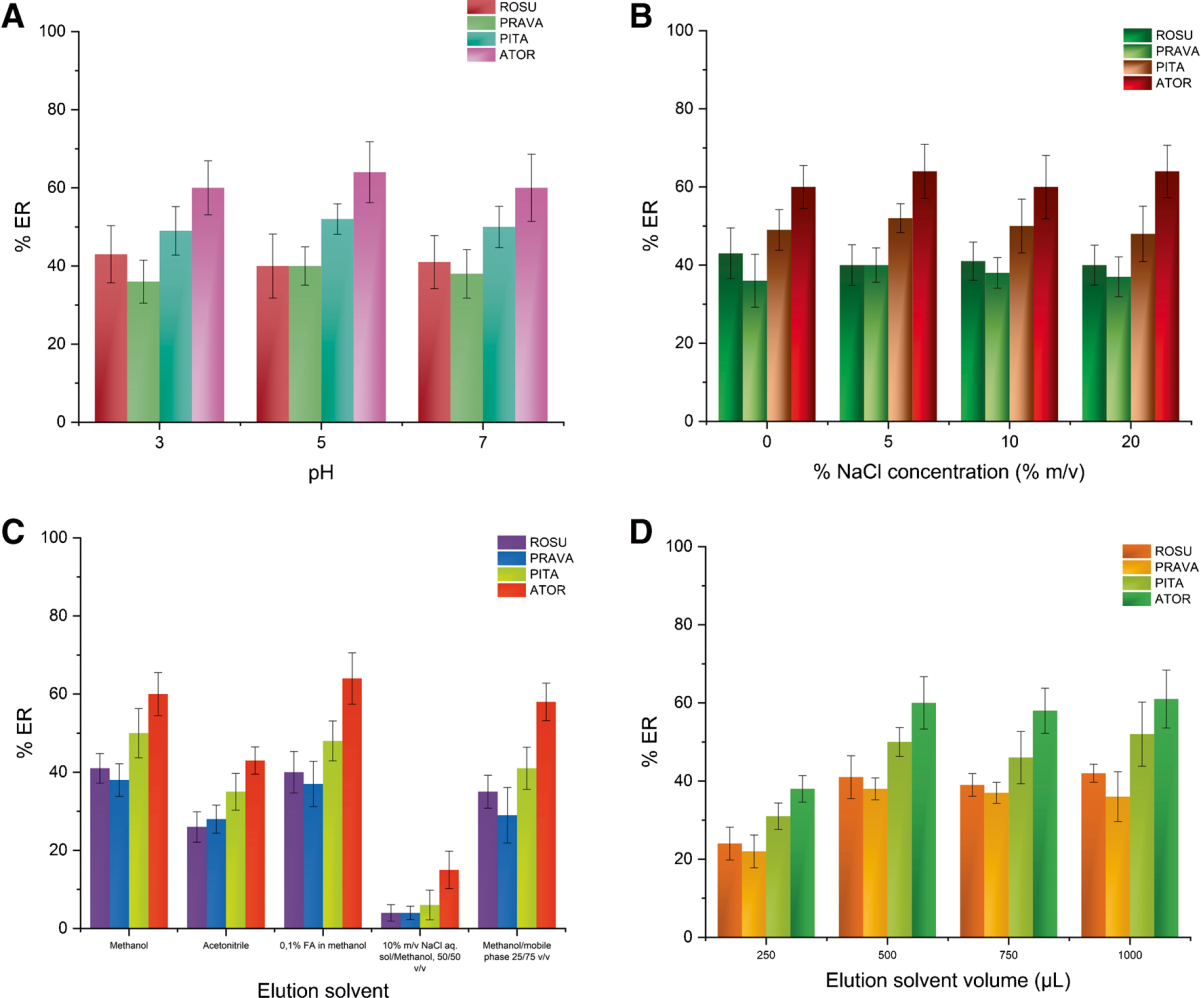
Effect of the (**A**) sample pH, (**B**) NaCl concentration (%*m*/*v*), (**C**) elution solvent type, and (**D**) elution solvent volume on the %ER of the studied analytes

The ionic strength and the pH value of the sample solution were examined as both chemical factors that may influence the extraction efficiency. The sample pH was studied in the range of 3–7 using appropriate phosphate buffers for pH adjustment. Similar %ER values were observed for all the target analytes within the examined pH range (Fig. 4A). To ensure handling simplicity and minimal sample pretreatment time prior to the CPME procedure, no pH adjustment was used in further experiments.

Afterward, the impact of the ionic strength was examined under different salinity concentrations (i.e., 0–20% NaCl *m*/*v*). Salt addition may decrease the solubility of compounds with intermediate polarity, favoring their transfer to the sol–gel sorbent. However, an increase in the viscosity of the solution might occur, reducing the mass transfer of the target analytes and thus decreasing their %ER values. As shown in Fig. 4B, the addition of NaCl did not have a profound impact on the extraction of statins, and thus, no adjustment of the ionic strength was chosen for the CPME protocol.

#### Optimization of the desorption step

The desorption conditions that could potentially affect the performance of the CPME method were evaluated. Five different eluents were examined including MeOH, ACN, 0.1% v/v formic acid in MeOH, MeOH:mobile phase (A) (25:75, v/v), and 10% m/v NaCl aqueous solution:MeOH, 50:50 v/v. Neat and acidified methanol (0.1% methanolic formic acid) provided the highest desorption efficiencies (Fig. 4C) for all the target analytes. In terms of simplicity, methanol was finally chosen as the eluent.

Accordingly, the effect of the eluent volume was studied between 250 and 1000 μL. An increase of the MeOH volume up to 500 μL had a positive impact on the extraction efficiency, while no further increase was observed using larger quantities (Fig. 4D). Thus, to ensure complete analyte desorption and reduced chemical consumption in following the principles of GAC [31], further experiments were conducted using 500 μL of MeOH during desorption.

Finally, the elution time was investigated in the range 2–15 min. In principle, the desorption time must be enough to provide sufficient contact of the sorbent with the eluent and to enable the desorption of the target analytes. At the same time, this time span must be short enough to result in a fast procedure. A time span of 5 min was sufficient for the complete desorption of the statins and was adopted for subsequent experiments (Supplementary Fig. S4).

### Comparison of the IL-based CPME platform with C_18_encapsulated media

Under optimum extraction conditions, the efficiency of the sol–gel Carbowax 20 M/ IL-based CPME platform was compared with C_18_ encapsulated media which is a widely used sorbent for this kind of analysis. The synthesis and characterization of sol–gel C_18_ CPME media has been reported elsewhere [16, 32]. Supplementary Fig. S5 summarizes the results of the comparative study. The %ER values of the sol–gel Carbowax 20 M/ IL-based CPME platform for all analytes were higher, demonstrating the superiority of this novel extraction phase toward the well-established C_18_ sorbent.

### Method validation and greenness evaluation

Method selectivity was examined through the analysis of blank pooled (*n* = 6) and spiked urine samples. Supplementary Fig. S6 shows a blank and a spiked HPLC–UV chromatogram of the pooled samples subjected to the CPME protocol. No interfering peaks from endogenous compounds were observed at the retention time of the target analytes and the ISTD, demonstrating method selectivity. The clean-up performance of the optimized CPME protocol was compared with the dilute-and-shoot methodology that is widely used in urine analysis. It can be seen in Supplementary Fig. S6 that the chromatogram after CPME is “cleaner” compared to the dilute-and-shoot approach. Thus, CPME can be considered an effective clean-up procedure that potentially increases the lifetime of the analytical column.

**Fig. 5:**
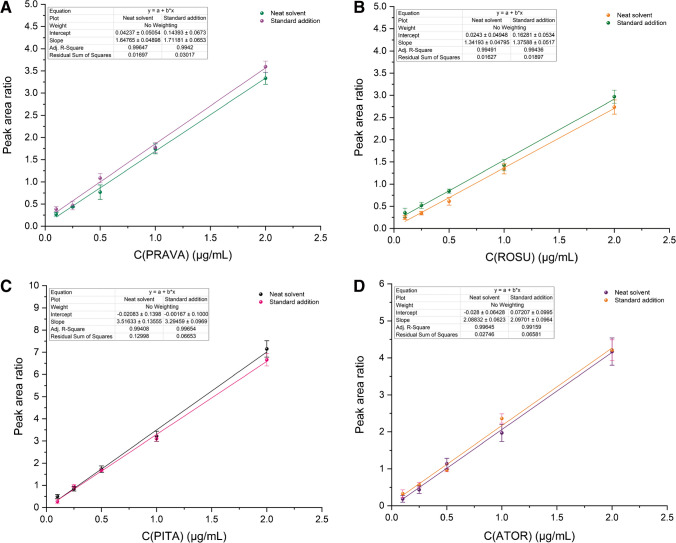
Calibration curves of the (**A**) PRAVA, (**B**) ROSU, (**C**) PITA, and (**D**) ATOR using the proposed CPME-HPLC–UV method. The HPLC instrumental parameters are described in the “Instrumentation” section

For method linearity, two individual calibration curves, i.e., a matrix-matched calibration curve and an aqueous calibration curve, were constructed within 0.10 μg mL^−1^ and 2.0 μg mL^−1^. In all cases, good linearity (*r* > 0.99) was observed (Fig. 5). Comparing the parallelism of the calibration curves the slope ratios ranged between 0.94 and 1.04, indicating the absence of matrix effect among the aqueous samples and the human urine for all analytes studied. For simplicity reasons, the aqueous calibration curve was finally chosen for quantitation. The LLOQ (*S*/*N* = 3) was 0.10 μg mL^−1^, while the LOD (*S*/*N* = 10) was calculated as 0.015 μg mL^−1^ for all drugs.

Table 1 shows the results for the accuracy and precision (i.e., intra-day and inter-day). As can be observed, the precision was better than 9.9% (intra-day) and 10.4% (inter-day study), respectively. The %RR for method accuracy was 83.4–106% for intra-day and 85.3–116%. In all cases, the results were within the acceptance limits of 80–120%.

**Table 1:** Intra-day, inter-day precision and accuracy of the developed CPME method for the determination of statins in human urine

Compound	Concentration level (μg mL^−1^)	Intra-day		Inter-day	
		Precision (%RSD)	Accuracy (RR^1^%)	Precision (%RSD)	Accuracy (RR %)
PRAVA	0.10 (LLOQ)	6.2	90.5	7.4	86.8
	0.25 (LQC)	2.3	103	3.5	96.3
	0.75 (MQC)	2.8	101	5.8	92.2
	2.0 (HQC)	1.8	86.4	0.9	86.6
POSU	0.10 (LLOQ)	6.7	93.9	8.7	91.9
	0.25 (LQC)	9.8	86.6	7.2	89.2
	0.75 (MQC)	4.8	106	6.4	95.1
	2.0 (HQC)	3.1	98.9	0.2	97.7
PITA	0.10 (LLOQ)	9.5	83.4	9.2	89.3
	0.25 (LQC)	9.9	105	8.7	99.2
	0.75 (MQC)	4.2	98.5	2.4	105
	2.0 (HQC)	0.4	92.4	0.5	95.7
ATOR	0.10 (LLOQ)	7.9	92.3	10.4	85.3
	0.25 (LQC)	4.8	96.7	1.2	116
	0.75 (MQC)	4.4	92.0	1.9	111
	2.0 (HQC)	0.9	105	0.6	106

^1^*RR*, relative recovery

The robustness of the method was studied by examining the effect of HPLC parameters on the resolution of the analytes. For this purpose, a Plackett–Burman design 2^7−4^ (including 3 center points) was built to examine the effects of the HPLC parameters, estimating only the main effects. The experimental domain was built using TIBCO Statistica software v. 13.3.0 (TIBCO software Inc., Palo Alto, CA, USA) and tabulated in Supplementary Table S7. Pareto charts (Supplementary Fig. S7) revealed that the buffer pH had a significant effect on the resolution between PRAVA, ROSU, and PITA, while the buffer concentration and pH are statistically significant in the rest of the compounds. However, as can be shown from the resolution data of Supplementary Table S7, the worst-case situation for resolution is the factor combination providing the lowest value of 1.98 which is acceptable according to the FDA Center for Drug Evaluation and Research (CDER) [33].

Analyte stability was evaluated in unprocessed urine at 0 h, 4 h, 8 h, and 24 h for samples stored at 25 °C, at 4 h, 8 h, and 24 h for samples stored at + 4 °C, and at 8 h, 24 h, and 48 h for samples stored at − 18 °C. A criterion of equal to or less than ± 15% deviation between the experimentally found concentration under each examined condition and the initial (or nominal) concentration in the biological sample was set. Under all the examined conditions, this criterion was met, proving analyte stability in the unprocessed human urine.

**Fig. 6:**
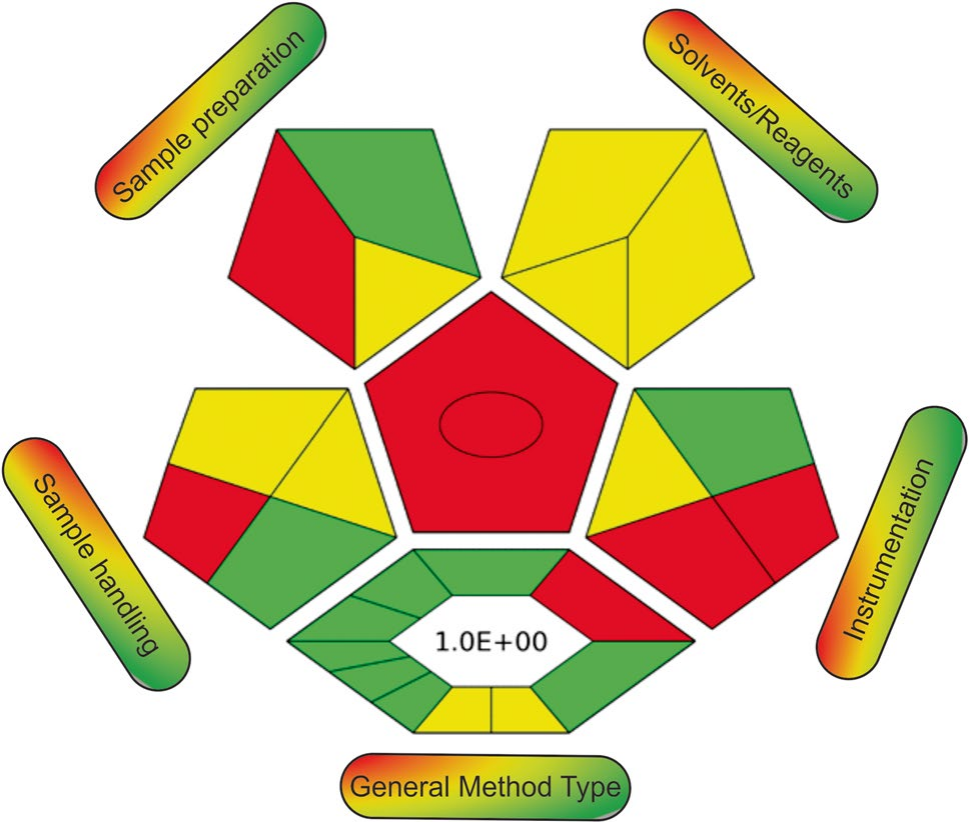
ComplexGAPI pictogram of the CPME-HPLC–UV method for the quantitation of statins in human urine

The green performance of the CPME-HPLC–UV method was evaluated by ComplexGAPI (Fig. 6) [34, 35]. As can be observed, the synthesis of the material complies with most criteria (green color). The chemical consumption is reduced since microextraction is employed, and the generation of waste during sample preparation is relatively low.

### Application to real samples

The new CPME-HPLC–UV method was used for the quantitation of the analytes in human urine samples in two volunteers. The samples were collected after 8 h from each patient after oral administration of EU-licensed PITA- and ROSU-containing tablets (2 mg PITA/tab and 5 mg ROSU/tab), respectively. No PITA and ROSU were detected in the analyzed sample with the developed method.

### Comparison with other studies

The proposed work was compared with other sorbent-based microextraction approaches reported in the literature for the determination of statins in biological fluids. As can be observed in Table 2, the RSD% values for the intra-day and inter-day precision of the proposed method were similar to those reported in refs. [36–38], lower compared to those reported in ref. [39], but higher compared to those reported in refs. [40, 41]. Thus, adequate precision was obtained. Better sensitivity was reported in other approaches; this fact can be attributed to the more sensitive instrumentation that was employed at most of them (i.e., HPLC–MS/MS and analogous systems). Indeed, the combination of the proposed CPME sample preparation scheme with more sensitive instrumentation could efficiently reduce the LOD and LOQ values obtained in this study. A disadvantage of this methodology compared to previously published sorbent-based microextraction methods is the relatively higher adsorption time, which can be attributed to the nature of CPME, which is an equilibrium-driven technique. However, this issue is eliminated by the inherent benefits of the sol–gel Carbowax 20 M/ IL composite-encapsulated CPME media. Through the introduction of the zwitterionic ionic liquid in addition to neutral, polar Carbowax 20 M polymer in the composite sorbent, no pH adjustment is required for the extraction of statins. At the same time, the porous nature of the polypropylene tube diminishes any need for protein precipitation prior to the microextraction, resulting in increased simplification of the sample preparation scheme. An additional benefit of CPME is the reusability of the microextraction media, in accordance with the principles of GAC and Green Sample Preparation [42], and the parallel sample handling using different capsules that enhances the overall sample throughput. All things considered, the final CPME method can serve as a powerful sample preparation technique for the extraction of statins from complex bioanalytical samples.

**Table 2:** Comparison of the proposed method with other sorbent-based microextraction approaches

Sample	Analyte	Sample preparation^1^	Sorbent	Adsorption time^2^ (min)	Instrumentation^3^	RSD%	LLOD (μg L^−1^)	Ref
Human serum	ROSU, ATOR, PITA, lovastatin and their metabolites	PP & MEPS	C_8_	NM	UHPLC-MS/MS	< 14.1	1	[37]
Human plasma	PRAVA, ATOR, fluvastatin	MEPS	C_18_	< 2	UHPLC-MS/MS	< 8.8	3.3–6.6	[34]
Human plasma, human urine, tablets	ROSU	MSPE	Magnetic nanoparticles modified with organic dendrimers containing methyl methacrylate and ethylene diamine	15	HPLC–UV	< 2.88	3.42	[38]
Human plasma	ATOR	PP & d-SPE	Ni metal–organic material modified with alumina nanoparticles	15	HPLC–UV	< 5	0.05	[39]
Human urine and human plasma	ROSU, ATOR	Micro SPE & SSME (human urine) PP & Micro SPE & SSME (human plasma)	Layered double hydroxide-coated magnetic nanoparticles	NM	HPLC–UV	< 9.3 (intra-day) < 9.7 (inter-day)	5–10	[35]
Human plasma	ATOR	PP & Micro SPE	C_18_	NM	HPLC–MS/MS	< 8.87	0.05	[36]
Human urine	PRAVA, ROSU, PITA, ATOR	CPME	Sol–gel Carbowax 20 M/3-[(3-Cholamidopropyl) dimethyl ammonio]-1-propanesulfonate composite	33	HPLC–UV	< 9.9 (intra-day) < 10.4 (inter-day)	15	**This study**

^1^*PP*, protein precipitation; *MEPS*, microextraction by packed sorbents; *MSPE*, magnetic solid-phase extraction; *d-SPE*, dispersive solid-phase extraction; *SPE*, solid-phase extraction; *SSME*, supramolecular solvent-based microextraction; *CPME*, capsule phase microextraction

^2^*NM*, not mentioned

^2^UHPLC-MS/MS, ultra high-performance liquid chromatography-tandem mass spectrometry; *HPLC–MS/MS*, high-performance liquid chromatography-tandem mass spectrometry; *HPLC–UV*, high-performance liquid chromatography-ultraviolet detection

## Conclusions

In this work, a task-specific sample preparation platform has been proposed using a sol–gel Carbowax 20 M/ IL-based absorbent for the isolation of statins from human urine. The hydrophobic and zwitterionic properties of the proposed CPME sorbent provided better extraction efficiency compared to the conventional C_18_ one. The proposed analytical scheme was effortless, fast, and economical and follows the requirements of GAC for step-integrating sample preparation methodologies. Due to the inherent properties of the CPME device’s built-in filtration capabilities, the extraction was performed directly in the unprocessed urine sample. Sample preparation workflow was systematically investigated and optimized. Under the optimum conditions, the developed analytical method presented satisfactory linearity, accuracy, and precision. The fabricated material can be reused at least 35 times. Although CPME is not automated, yet it is capable of being automated, offering high sample throughput. All things considered, the sample preparation technique is an effective and simple tool that can be used for the determination of statins in urine. Obviously, it has the potential to be a suitable bioanalytical tool, and thus, the expansion of the application of CPME in other bio-samples would be beneficial.

### Supplementary Information

Below is the link to the electronic supplementary material.

Supplementary file1 (DOCX 2168 KB)

## Data Availability

Data will be made available on request.
