# Supplementary material for: Fabricating a designer capsule phase microextraction platform based on sol–gel Carbowax 20M-zwitterionic ionic liquid composite sorbent for the extraction of lipid-lowering drugs from human urine samples
Source: Mikrochim Acta. 2023 Oct 5;190(11):428. doi: 10.1007/s00604-023-05998-3 (PMC10556171; doi:10.1007/s00604-023-05998-3)
Supplement: Supplementary file 1 — Supplementary file1 (DOCX 2168 KB) [file 604_2023_5998_MOESM1_ESM.docx]

**Electronic Supplementary Material**

**Fabricating a designer capsule phase microextraction platform based on sol-gel Carbowax 20M-zwitterionic ionic liquid composite sorbent for the extraction of lipid-lowering drugs from human urine samples**

Argyroula Kechagia^1^, Natalia Manousi^1^, Abuzar Kabir*^2^, Kenneth G. Furton^2^, Constantinos K. Zacharis*^1^

*^1^Laboratory of Pharmaceutical Analysis, Department of Pharmaceutical Technology, School of Pharmacy, Aristotle University of Thessaloniki, 54124 Thessaloniki, Greece*

*^2^International Forensic Research Institute, Department of Chemistry and Biochemistry, Florida International University, Miami, FL, 33131, USA*

|  |  |
| --- | --- |

*Corresponding author

Constantinos K. Zacharis

Assistant Professor

Laboratory of Pharmaceutical Analysis, School of Pharmacy,

Aristotle University of Thessaloniki (AUTh),

GR-54124, Greece

Tel: +30 2310997663

E-mail: [czacharis@pharm.auth.gr](mailto:czacharis@pharm.auth.gr)

*Corresponding author

Abuzar Kabir

Research Associate Professor,

International Forensic Research Institute,

Department of Chemistry and Biochemistry,

Florida International University, Miami, FL, USA

Tel: 3053482396

E-mail: [akabir@fiu.edu](mailto:akabir@fiu.edu)

**
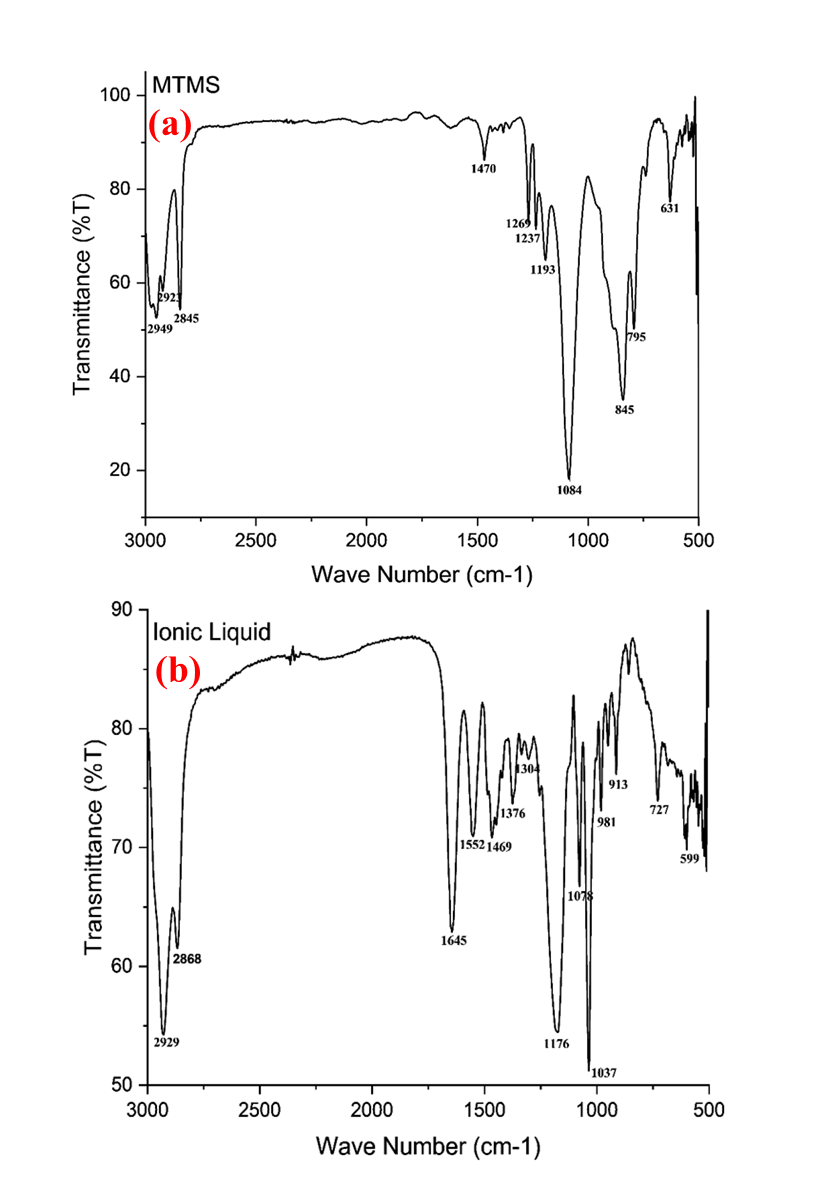
**

**Figure S1**. FT-IR spectra of (a) Methyl trimethoxysilane; (b) 3-[(3-cholamidopropyl) dimethyl ammonio]-1-propanesulfonate ionic liquid

**
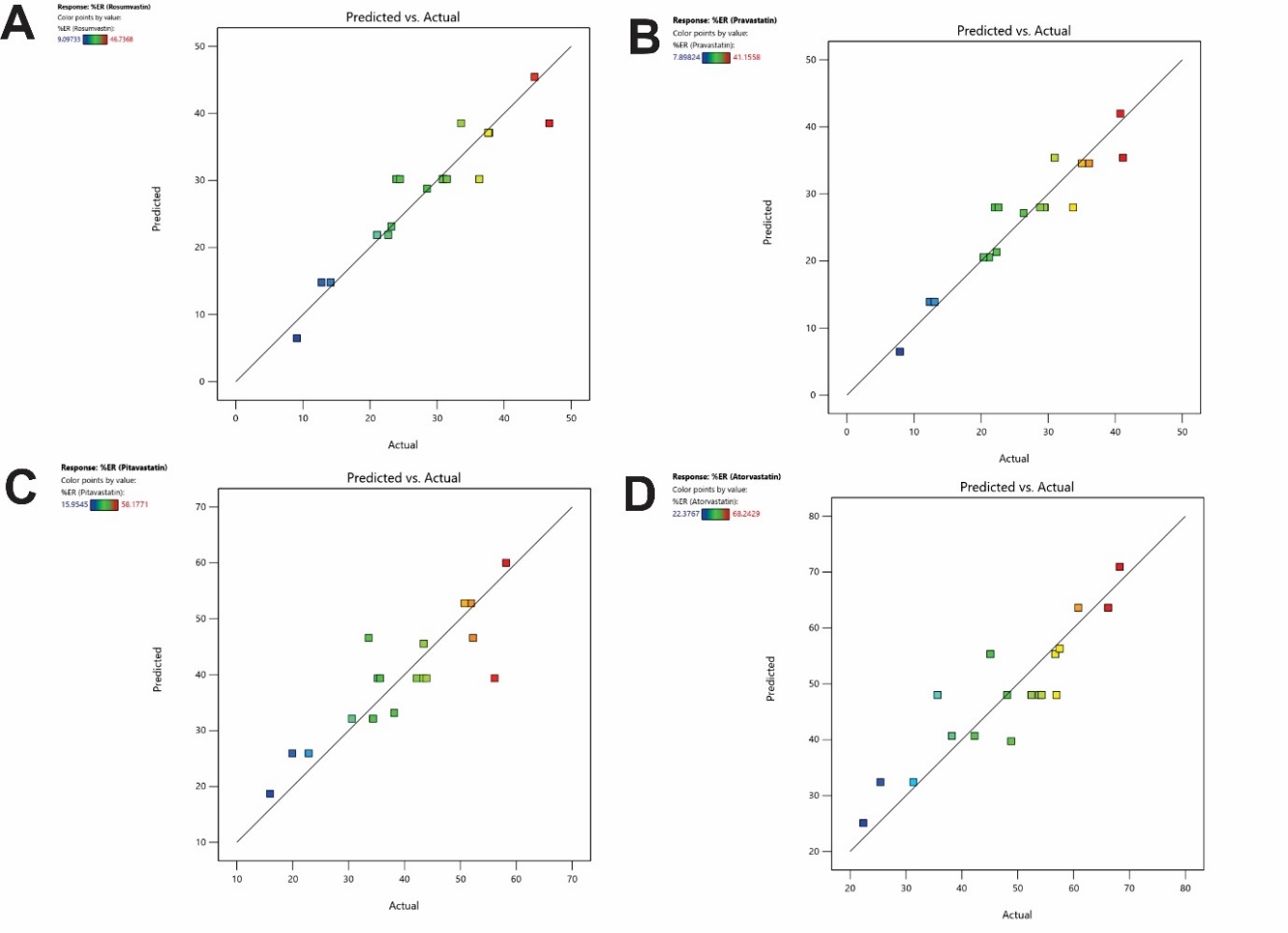
**

**Figure S2.** Residuals vs predicted plots for A) ROSU, B) PRAVA, C) PITA and D) ATOR.


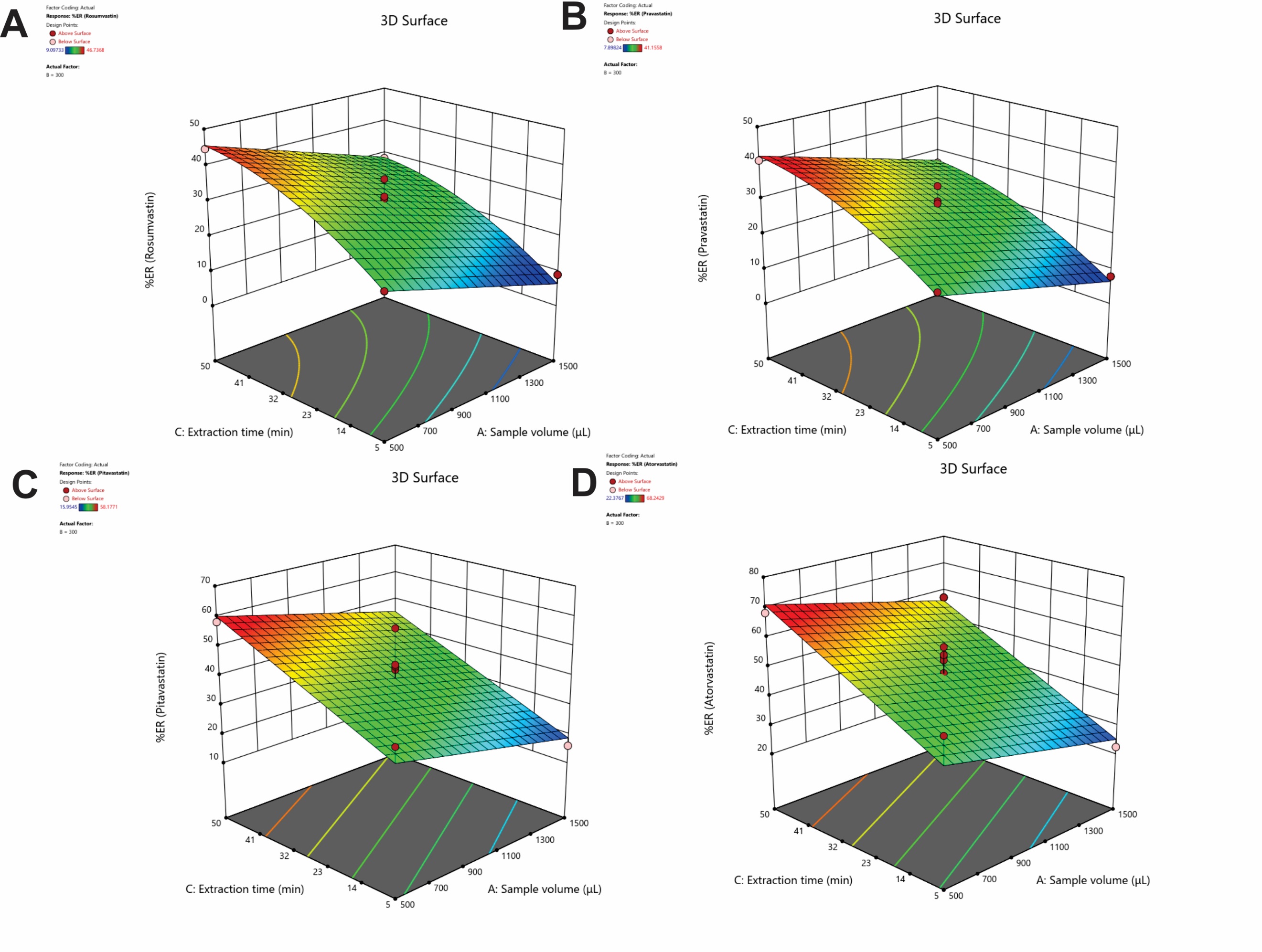


**Figure S3.** 3D plots showing the effects of extraction time and sample volume on the %ER of A) ROSU, B) PRAVA, C) PITA and D) ATOR.

**
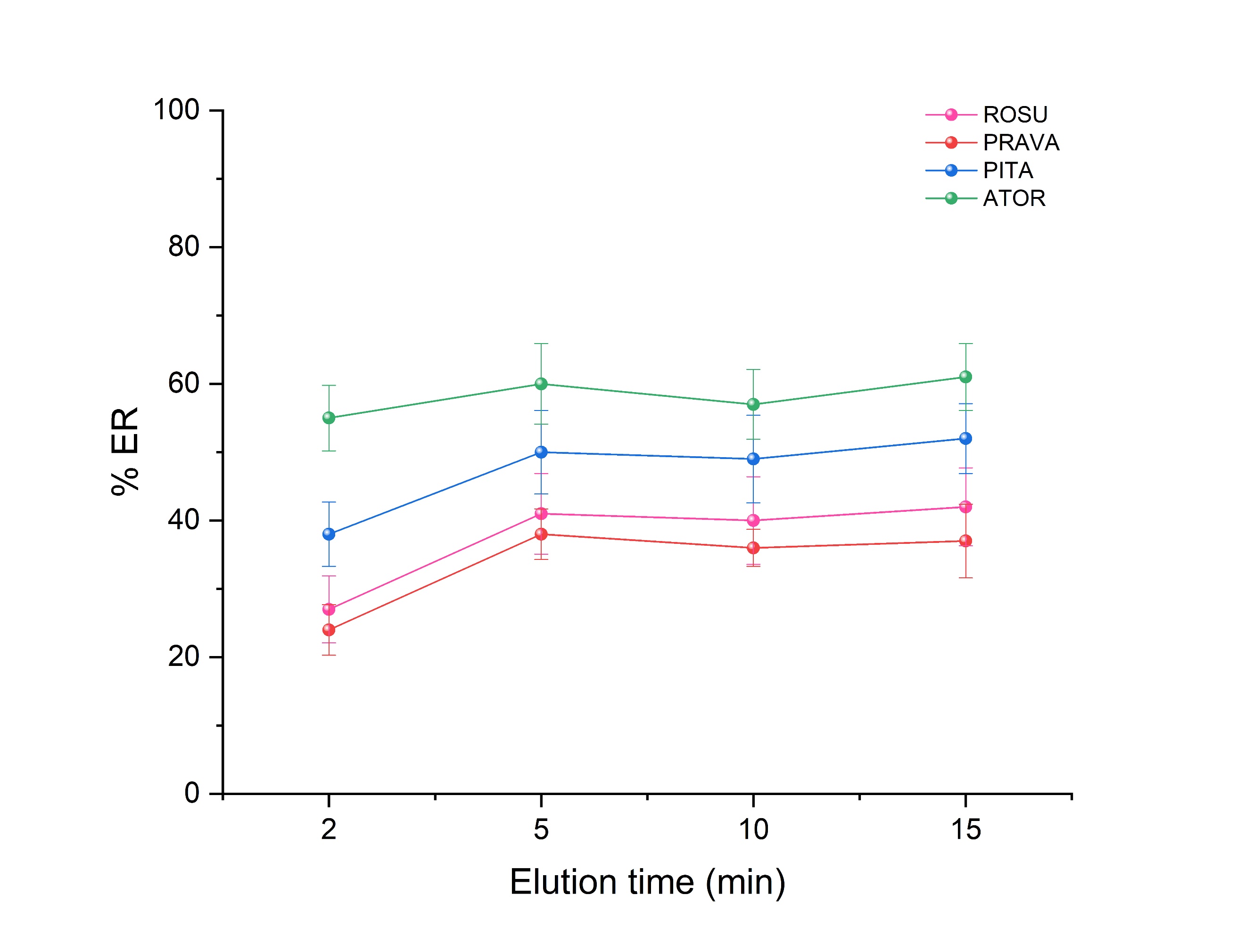
**

**Figure S4.** Effect of the elution time on the % ER of A) ROSU, B) PRAVA, C) PITA and D) ATOR.


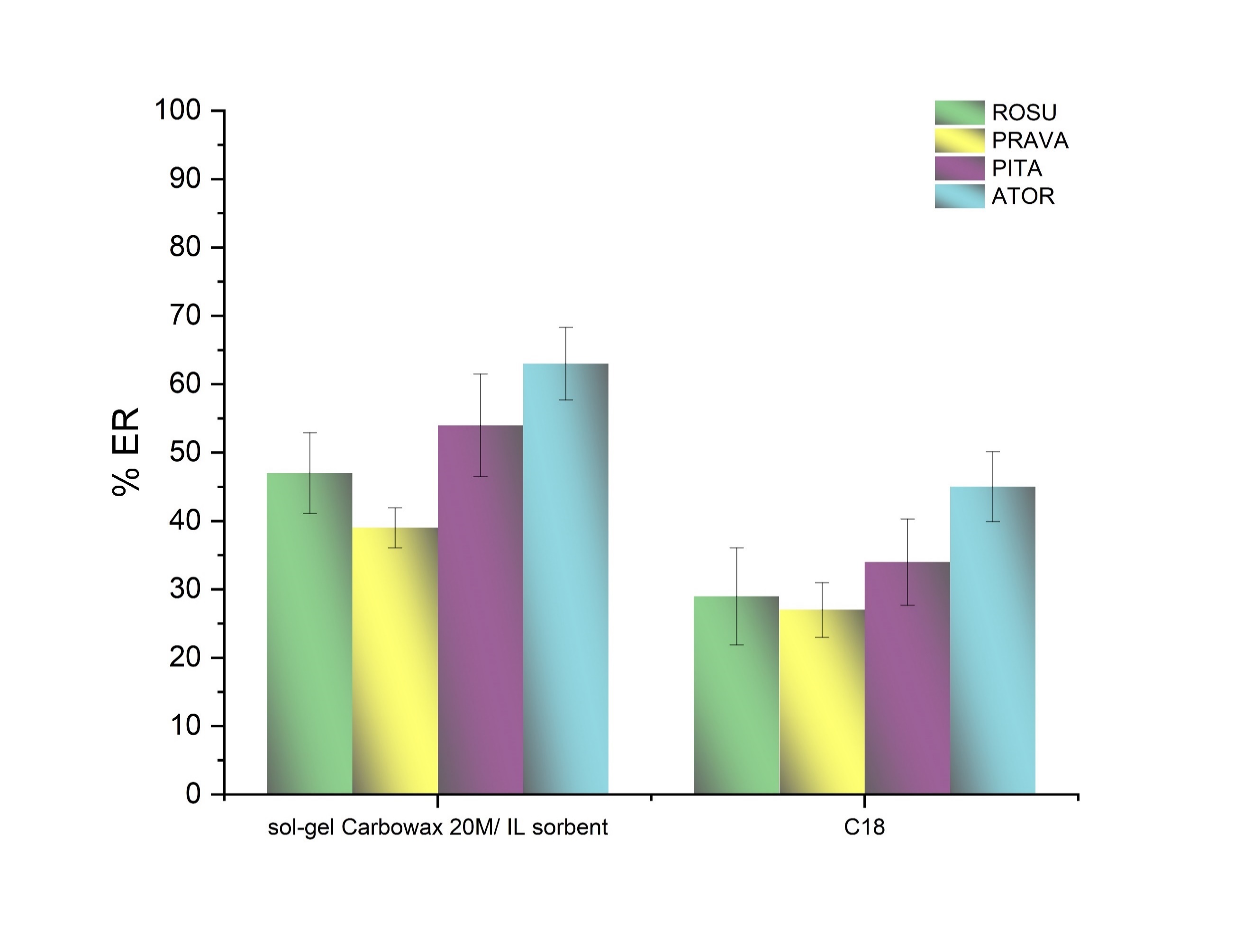


**Figure S5.** Comparison of the A) sol-gel Carbowax 20M/ IL sorbent and B) conventional C_18_ CPME sorbents on the % extraction recovery of the studied drugs.


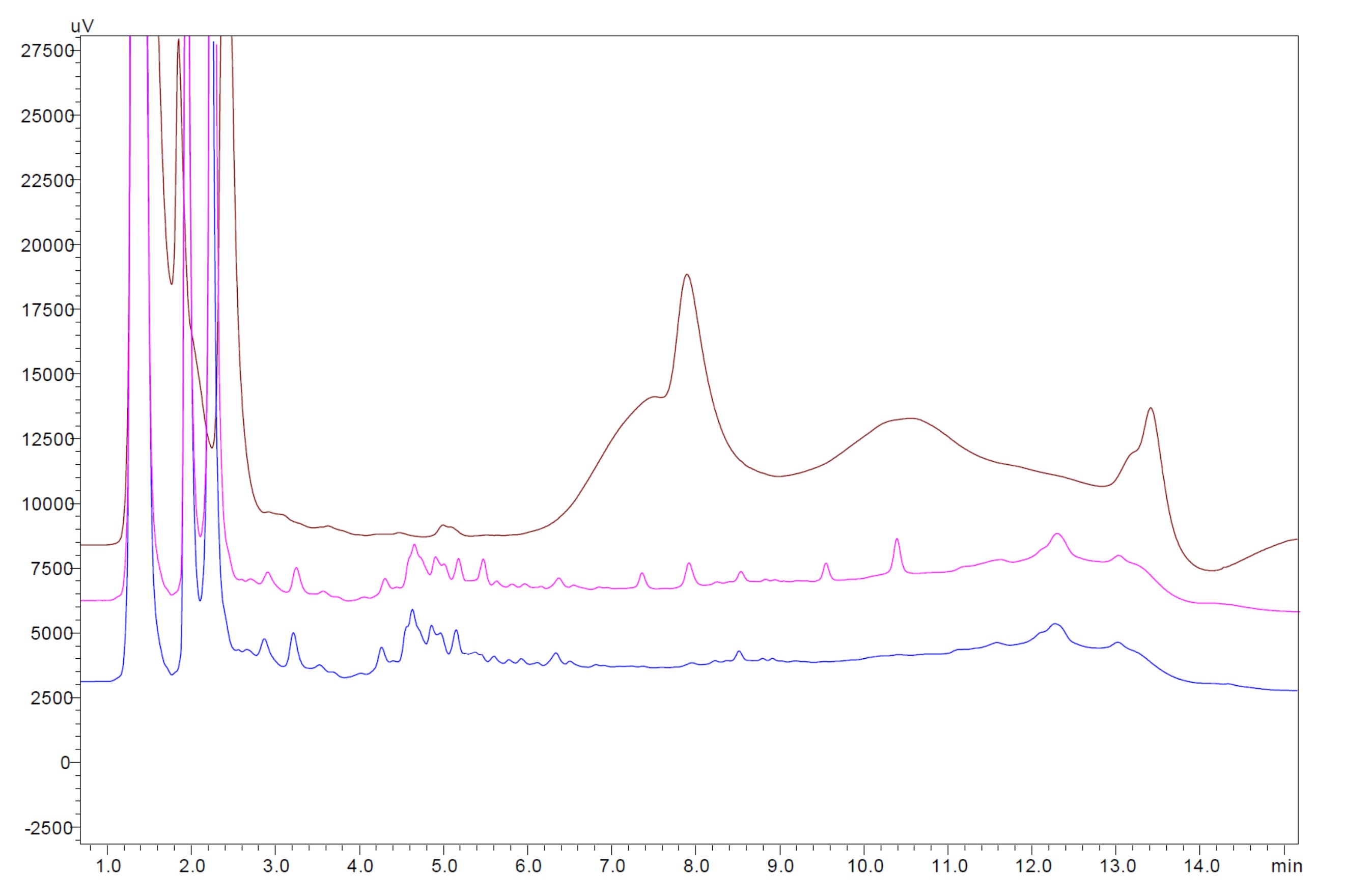


ATOR

C

B

A

ISTD

ROSU

PITA

PRAVA

**Figure S6.** Representative overlaid HPLC-UV chromatograms of the analysis of (A) drug-free pooled urine sample (*n* = 6) after CPME, (B) spiked with the target analytes and ISTD at LLOQ level after CPME and (C) drug-free urine sample (5-fold diluted) without CPME.

**
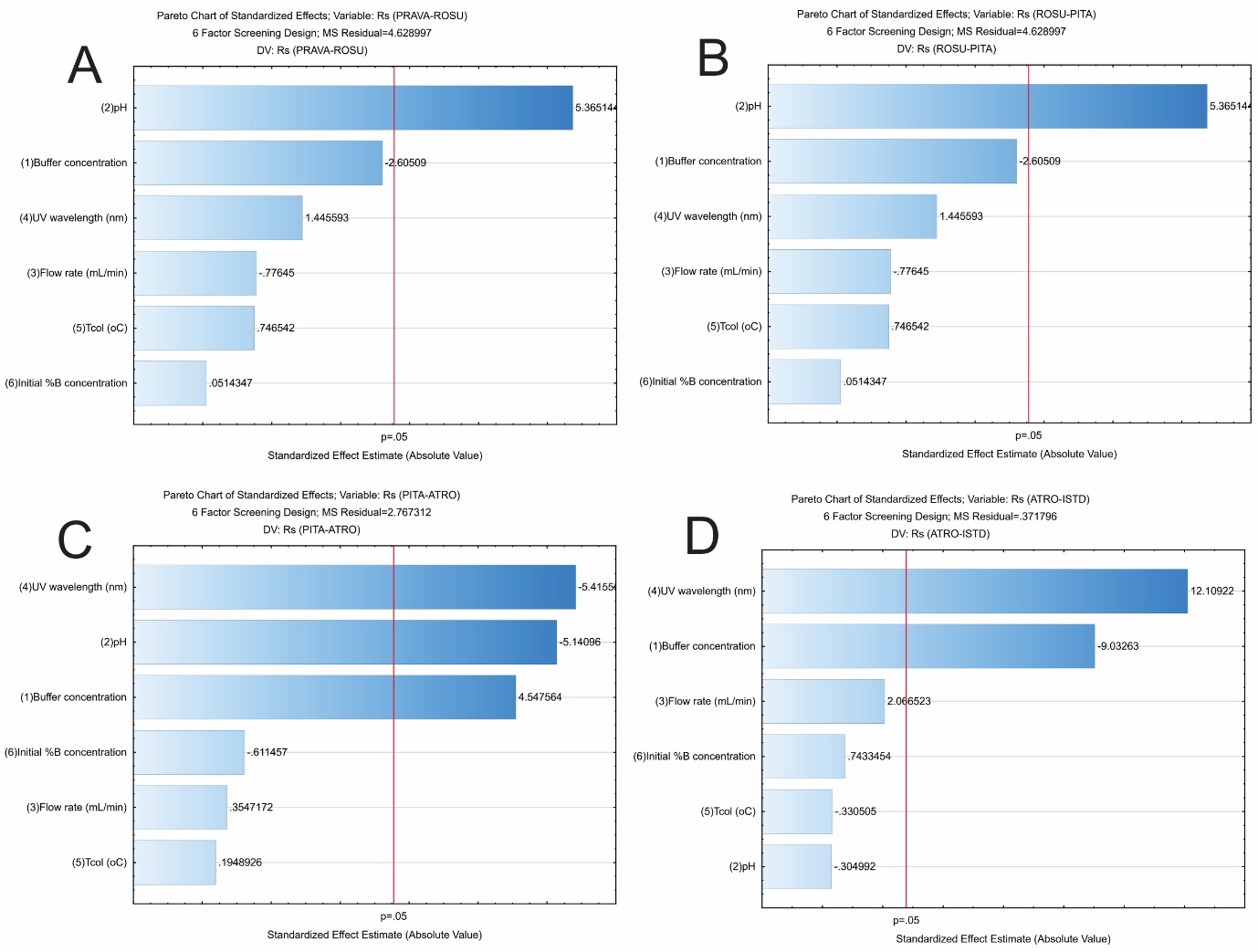
**

**Figure S7.** Pareto charts for the robustness test of HPLC instrumental parameters. Responses: A) R_s(PRAVA-ROSU)_, Β) R_s(ROSU-PITA)_, C) R_s(PITA-ATOR)_ and D) R_s(ATOR-ISTD)_.

**Table S1**. Chemical structures and relevant physicochemical properties of the studied statins.

| **Compound** | **Chemical Structure** | **Chemical Formula** | **pK_a_** | **LogK_ow_** |
| --- | --- | --- | --- | --- |
| Atorvastatin |  | C_33_H_35_FN_2_O_5_ | 4.5 | 6.36 |
| Pitavastatin |  | C_25_H_24_FNO_4_ | 4.3 | 4.82 |
| Rosuvastatin |  | C_22_H_28_FN_3_O_6_S | 4.0 | 2.48 |
| Pravastatin |  | C_23_H_36_O_7_ | 4.2 | 2.18 |

**Table S2**. Experimental BBD domain for the optimization of CPME parameters.

|  |  |  |  | **%ER** | | | |
| --- | --- | --- | --- | --- | --- | --- | --- |
| **Standard Run** | **Sample volume (μL)**  **(Factor A)** | **Stirring rate (rpm)**  **(Factor B)** | **Extraction time (min)**  **(Factor C)** | **ROSU** | **PRAVA** | **PITA** | **ATOR** |
| 1 | 500 | 300 | 5 | 23.2 | 22.3 | 38.2 | 48.8 |
| 2 | 1000 | 300 | 27.5 | 24.5 | 22.6 | 35.6 | 35.6 |
| 3 | 1500 | 500 | 27.5 | 21.1 | 20.4 | 30.6 | 38.2 |
| 4 | 500 | 100 | 27.5 | 33.6 | 30.9 | 33.6 | 56.7 |
| 5 | 1000 | 500 | 50 | 37.6 | 35.1 | 50.7 | 66.2 |
| 6 | 1000 | 100 | 5 | 12.8 | 12.3 | 19.9 | 25.4 |
| 7 | 1500 | 300 | 50 | 28.5 | 26.3 | 43.4 | 57.5 |
| 8 | 1000 | 500 | 5 | 14.1 | 13.1 | 22.8 | 31.3 |
| 9 | 1000 | 100 | 50 | 37.7 | 36.1 | 51.9 | 60.8 |
| 10 | 1500 | 100 | 27.5 | 22.7 | 21.2 | 34.4 | 42.3 |
| 11 | 1000 | 300 | 27.5 | 31.4 | 28.8 | 43.9 | 56.9 |
| 12 | 500 | 300 | 50 | 44.5 | 40.8 | 58.2 | 68.2 |
| 13 | 1000 | 300 | 27.5 | 23.9 | 22.1 | 35.2 | 48.1 |
| 14 | 500 | 500 | 27.5 | 46.7 | 41.1 | 52.3 | 45.1 |
| 15 | 1000 | 300 | 27.5 | 30.9 | 29.5 | 42.2 | 52.4 |
| 16 | 1000 | 300 | 27.5 | 30.8 | 29.4 | 43.3 | 54.3 |
| 17 | 1500 | 300 | 5 | 9.1 | 7.9 | 15.9 | 22.4 |
| 18 | 1000 | 300 | 27.5 | 36.3 | 33.7 | 56.1 | 53.9 |

**Table S3**. Analysis of variance (ANOVA) for BBD for ER% of ROSU.

| **Source** | **Sum of Squares** | **df** | **Mean Square** | ***F*-value** | ***p*-value** |  |
| --- | --- | --- | --- | --- | --- | --- |
| **Model** | 1631.22 | 3 | 543.74 | 34.89 | < 0.0001 | significant |
| A-Sample volume | 555.38 | 1 | 555.38 | 35.64 | < 0.0001 |  |
| C-Extraction time | 995.61 | 1 | 995.61 | 63.89 | < 0.0001 |  |
| C² | 80.23 | 1 | 80.23 | 5.15 | 0.0396 |  |
| **Residual** | 218.17 | 14 | 15.58 |  |  |  |
| Lack of Fit | 108.00 | 9 | 12.00 | 0.5446 | 0.7982 | not significant |
| Pure Error | 110.17 | 5 | 22.03 |  |  |  |
| **Cor Total** | 1849.38 | 17 |  |  |  |  |

**Table S4**. Analysis of variance (ANOVA) for BBD for ER% of PRAVA.

| **Source** | **Sum of Squares** | **df** | **Mean Square** | ***F*-value** | ***p*-value** |  |
| --- | --- | --- | --- | --- | --- | --- |
| **Model** | 1357.08 | 3 | 452.36 | 38.13 | < 0.0001 | significant |
| A-Sample volume | 440.99 | 1 | 440.99 | 37.17 | < 0.0001 |  |
| C-Extraction time | 853.69 | 1 | 853.69 | 71.96 | < 0.0001 |  |
| C² | 62.39 | 1 | 62.39 | 5.26 | 0.0378 |  |
| **Residual** | 166.08 | 14 | 11.86 |  |  |  |
| Lack of Fit | 64.49 | 9 | 7.17 | 0.3527 | 0.9171 | not significant |
| Pure Error | 101.58 | 5 | 20.32 |  |  |  |
| **Cor Total** | 1523.15 | 17 |  |  |  |  |

**Table S5**. Analysis of variance (ANOVA) for BBD for ER% of PITA.

| **Source** | **Sum of Squares** | **df** | **Mean Square** | ***F*-value** | ***p*-value** |  |
| --- | --- | --- | --- | --- | --- | --- |
| **Model** | 1860.30 | 2 | 930.15 | 21.24 | < 0.0001 | significant |
| A-Sample volume | 417.94 | 1 | 417.94 | 9.55 | 0.0075 |  |
| C-Extraction time | 1442.36 | 1 | 1442.36 | 32.94 | < 0.0001 |  |
| **Residual** | 656.75 | 15 | 43.78 |  |  |  |
| Lack of Fit | 368.39 | 10 | 36.84 | 0.6388 | 0.7448 | not significant |
| Pure Error | 288.36 | 5 | 57.67 |  |  |  |
| **Cor Total** | 2517.05 | 17 |  |  |  |  |

**Table S6**. Analysis of variance (ANOVA) for BBD for ER% of ATOR.

| **Source** | **Sum of Squares** | **df** | **Mean Square** | ***F*-value** | ***p*-value** |  |
| --- | --- | --- | --- | --- | --- | --- |
| **Model** | 2375.53 | 2 | 1187.77 | 29.49 | < 0.0001 | significant |
| A-Sample volume | 428.31 | 1 | 428.31 | 10.64 | 0.0053 |  |
| C-Extraction time | 1947.22 | 1 | 1947.22 | 48.35 | < 0.0001 |  |
| **Residual** | 604.08 | 15 | 40.27 |  |  |  |
| Lack of Fit | 307.23 | 10 | 30.72 | 0.5175 | 0.8244 | not significant |
| Pure Error | 296.85 | 5 | 59.37 |  |  |  |
| **Cor Total** | 2979.61 | 17 |  |  |  |  |

**Table S7**. Plans of experiments generated by Plackett-Burman design.

| **Run No** | **C_Buffer_ (mM)** | **Buffer pH** | **Flow rate (mL min^-1^)** | **UV wavelength (nm)** | **T_col_ (^o^C)** | **Initial %B concentration** | **Dummy1** | **R_s(PRAVA-ROSU)_** | **R_s(ROSU-PITA)_** | **R_s(PITA-ATOR)_** | **R_s(ATOR-ISTD)_** |
| --- | --- | --- | --- | --- | --- | --- | --- | --- | --- | --- | --- |
| 1 | 22 | 3.2 | 0.95 | 238 | 28 | 24 | -1 | 12.39 | 2.39 | 10.57 | 4.60 |
| 2 | 22 | 3.2 | 1.05 | 238 | 32 | 26 | 1 | 13.47 | 2.99 | 10.99 | 5.47 |
| 3 | 18 | 3.2 | 0.95 | 236 | 32 | 24 | 1 | 16.34 | 4.32 | 8.45 | 5.51 |
| 4 | 22 | 2.8 | 1.05 | 236 | 32 | 24 | -1 | 1.98 | 15.46 | 6.23 | 7.69 |
| 5 | 22 | 2.8 | 0.95 | 236 | 28 | 26 | 1 | 3.15 | 14.76 | 5.16 | 6.57 |
| 6 | 20 | 3.0 | 1.00 | 237 | 30 | 25 | 0 | 12.5 | 4.04 | 9.66 | 4.75 |
| 7 | 20 | 3.0 | 1.00 | 237 | 30 | 25 | 0 | 12.47 | 4.1 | 9.59 | 4.85 |
| 8 | 18 | 2.8 | 0.95 | 238 | 32 | 26 | -1 | 9.41 | 2.60 | 14.77 | 5.06 |
| 9 | 18 | 2.8 | 1.05 | 238 | 28 | 24 | 1 | 8.06 | 4.19 | 14.84 | 4.35 |
| 10 | 20 | 3.0 | 1.00 | 237 | 30 | 25 | 0 | 12.43 | 4.09 | 9.66 | 4.93 |
| 11 | 18 | 3.2 | 1.05 | 236 | 28 | 26 | -1 | 13.05 | 3.12 | 10.46 | 4.38 |
